# Supplementary figures and images for: Comparative Genomic Analysis Uncovers the Chloroplast Genome Variation and Phylogenetic Relationships of Camellia Species
Source: Biomolecules. 2022 Oct 13;12(10):1474. doi: 10.3390/biom12101474 (PMC9599789; doi:10.3390/biom12101474)

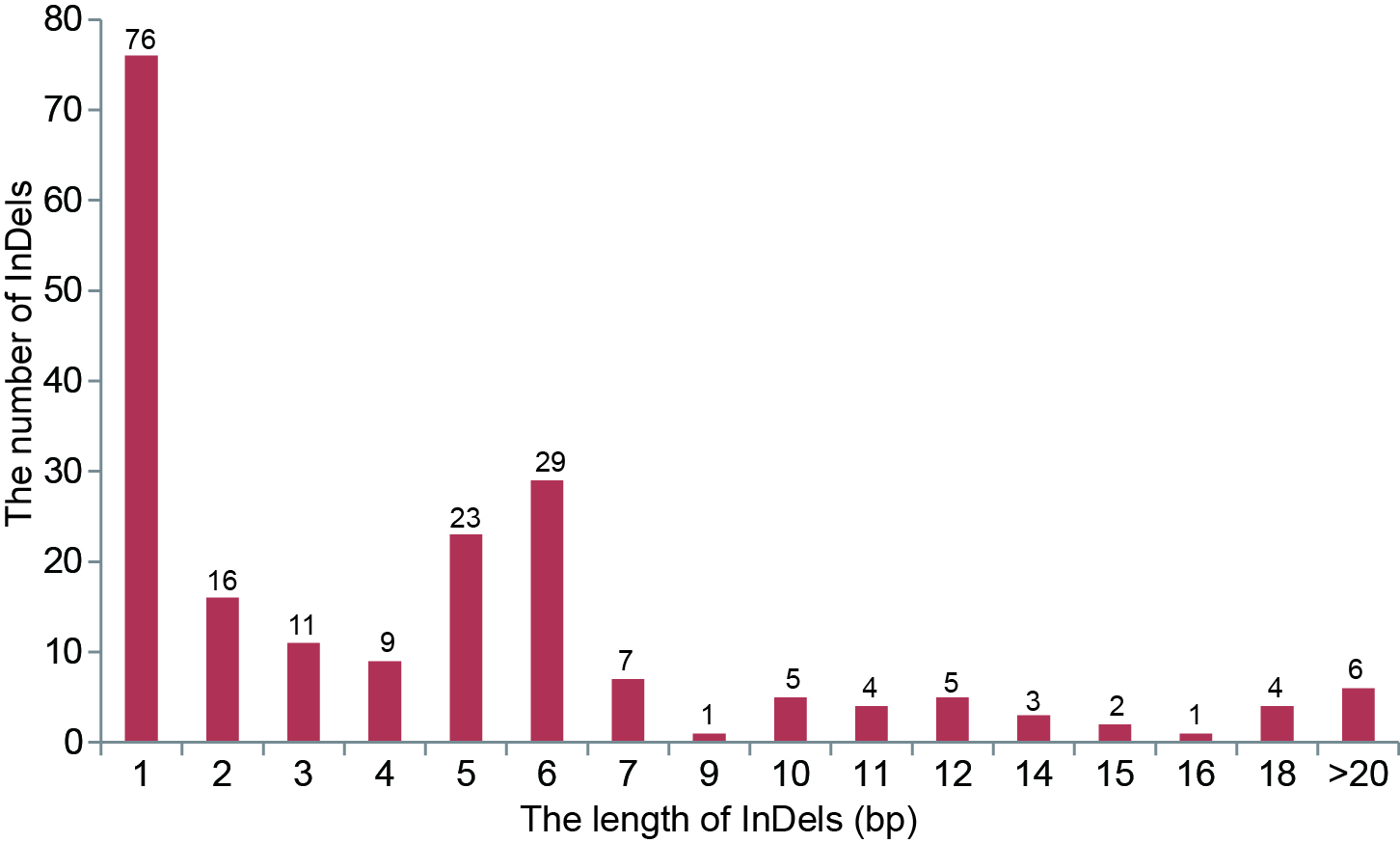

Supplement: Supplementary file 1 [file biomolecules-12-01474-s001.zip › Figure S1 the distribution of InDel types in the 12 sequenced Camellia cp genomes.jpg]
